# A Randomized Controlled Trial of Glucose versus Amylase Resistant Starch Hypo-Osmolar Oral Rehydration Solution for Adult Acute Dehydrating Diarrhea

**DOI:** 10.1371/journal.pone.0001587

**Published:** 2008-02-13

**Authors:** Balakrishnan S. Ramakrishna, Venkataraman Subramanian, Vivek Mohan, Bendon K. Sebastian, Graeme P. Young, Michael J. Farthing, Henry J. Binder

**Affiliations:** 1 Wellcome Trust Research Laboratory, Christian Medical College, Vellore, India; 2 Department of Medicine, Flinders University, Bedford Park, Adelaide, South Australia, Australia; 3 St. George's, University of London, Tooting, London, United Kingdom; 4 Department of Internal Medicine, Yale University School of Medicine, New Haven, Connecticut, United States of America; Yale University School of Medicine, United States of America

## Abstract

**Background:**

Reduction of gross diarrhea rate in excess of that seen over time with intravenous therapy and appropriate antibiotics is not usually achieved by oral glucose-electrolyte rehydration therapy for cholera and cholera-like diarrheas.

**Methodology and Principal Findings:**

This prospective randomized clinical trial at a tertiary referral hospital in southern India was undertaken to determine whether amylase resistant starch, substituting for glucose in hypo-osmolar oral rehydration solution, would reduce diarrhea duration and weight in adults with acute severe dehydrating diarrhea. 50 adult males with severe watery diarrhea of less than three days' duration and moderate to severe dehydration were randomized to receive hypo-osmolar ORS (HO-ORS) or HO-ORS in which amylase resistant high amylose maize starch 50g/L substituted for glucose (HAMS-ORS). All remaining therapy followed standard protocol. Duration of diarrhea (ORS commencement to first formed stool) in hours was significantly shorter with HAMS-ORS (median 19, IQR 10-28) compared to HO-ORS (median 42, IQR 24-50) (Bonferroni adjusted P, P_adj_<0.001). Survival analysis (Kaplan-Meier) showed faster recovery from diarrhea in the HAMS-ORS group (P<0.001, log rank test). Total diarrhea fecal weight in grams (median, IQR) was not significantly lower in the HAMS-ORS group (2190, 1160-5635) compared to HO-ORS (5210, 2095-12190) (P_adj_ = 0.08). However, stool weight at 13-24 hours (280, 0-965 vs. 1360, 405-2985) and 25–48 hours (0, 0-360 vs. 1080, 55-3485) were significantly lower in HAMS-ORS compared to HO-ORS group (P_adj_ = 0.048 and P = 0.012, respectively). ORS intake after first 24 hours was lower in the HAMS-ORS group. Subgroup analysis of patients with culture isolates of *Vibrio cholerae* indicated similar significant differences between the treatment groups.

**Conclusions:**

Compared to HO-ORS, HAMS-ORS reduced diarrhea duration by 55% and significantly reduced fecal weight after the first 12 hours of ORS therapy in adults with cholera-like diarrhea.

**Trial Registration:**

Current Controlled Trials ISRCTN72841333

## Introduction

Cholera and acute dehydrating diarrhea continue to be a major cause of morbidity and mortality in resource poor settings. Oral rehydration therapy using glucose salt oral rehydration solution (ORS) is the cornerstone of treatment [1], and exploits the fact that glucose-dependent sodium absorption in the small intestine remains intact even as intestinal secretion continues [2]. The iso-osmolar (compared to plasma) glucose-based oral rehydration solution originally recommended by the World Health Organization did not shorten the duration or severity of diarrhea and sometimes paradoxically increased diarrhea volume, leading to poor acceptance of ORS in many communities [3]. Studies in experimental diarrhea indicated that reducing the glucose and sodium concentration of ORS, resulting in osmolarity below that of plasma (hypo-osmolar ORS), increased small intestinal water absorption compared to the iso-osmolar ORS [4]. A meta-analysis of the resulting clinical trials concluded that hypo-osmolar ORS reduced diarrhea in non-cholera illness by 20% compared to conventional ORS [5]. In 2003, the World Health Organization recommended that ORS osmolarity and sodium content be reduced [6], [7].

The colon is capable of absorbing a considerable amount of salt and water under conditions of stress as occurs in secretory diarrhea [8]. Complex carbohydrates such as rice powder have been used in place of glucose in ORS and have been widely accepted [9]. It has been hypothesized that complex carbohydrate provides glucose at the mucosal interface without introducing an osmotic penalty [10]. However, an alternative explanation is that a proportion of complex carbohydrate passes into the colon to be fermented to short chain fatty acids (SCFA), which are known to stimulate sodium and water absorption from the secreting colon [11]. High amylose maize starch (HAMS), obtained from a particular variety of corn, contains both an amylase-digestible component that provides glucose by enzymatic digestion in the small intestine (without osmotic penalty) and a high proportion of amylase-resistant starch that is not absorbed in the small intestine and therefore reaches the colon [12]. HAMS, given orally, increased fecal SCFA concentrations in healthy volunteers [13]. In adults with cholera, HAMS significantly reduced diarrhea duration and fecal weight when added to conventional ***iso-osmolar*** glucose ORS [14].

We hypothesized that HAMS would reduce stool output and diarrhea duration in cholera when substituted for glucose in a low sodium hypo-osmolar ORS, thereby achieving what the hypo-osmolar ORS *per se* does not achieve in cholera. This present study in adults was designed to determine whether ORS efficacy could be further increased by substituting HAMS for glucose in ***hypo-osmolar*** ORS, an intervention that would have the consequences of both further lowering osmolarity and providing carbohydrate substrate to the colon for SCFA generation. A similar trial in chidren under the age of five, with non-cholera diarrhea, was registered along with this trial in adults but will be completed by a different team and reported separately.

## Methods

The protocol for this trial and supporting CONSORT checklist are available as supporting information; see Checklist S1 and Protocol S1.

### Participants

Consecutive patients presenting to the Emergency Services of the Christian Medical College between May 2003 and June 2005, who met the entry criteria and consented to participate, were referred to the study medical officers. Patients were considered for eligibility if they were males aged between 12–65 years with watery diarrhea of less than 3 days duration, and moderate to severe dehydration [15] at the time of presentation to the Emergency Services. Patients with bloody diarrhea as well as those with co-existing medical illness, including malignancy and clinical cardiopulmonary or liver disease, were excluded. Women were excluded by convention because of difficulty in separating diarrhea stool from urine. Patients were resuscitated in the Emergency Services by intravenous infusion with full strength Hartmann's Ringer's lactate solution, 100 ml/kg over four hours. Doxycycline 300 mg in a single oral dose was given as soon as the patient was capable of oral intake. Both these measures represent the current standard of care for management of cholera at this institution. The patient was admitted into the study ward after obtaining consent.

### Ethics

Individual participants gave written informed consent. The study protocol and consent forms were approved by the Institutional Review Board of the Christian Medical College, Vellore.

### Interventions

After admission into the study, patients were randomly allocated to receive one of two ORSs, either hypo-osmolar ORS (HO-ORS) or HO-ORS in which glucose was completely replaced by 50 g/L high amylose maize starch (National Starch, USA) (HAMS-ORS). As can be seen from the composition of the two ORSs (Table 1), there was no glucose in HAMS-ORS and the osmolarity of this ORS was lower than of HO-ORS. Both ORSs were packaged in sachets in quantities suitable for reconstitution to 200 ml with water. Instructions regarding ORS reconstitution and intake, and instructions regarding urine and stool collection were given to the patient and his attendant. ORS was administered in a dose of 200 ml per hour and 200 ml after each loose stool. Intake of water and other fluids was allowed and a standard Indian diet was immediately allowed. Each stool was separately collected in a bucket lined with a plastic bag, and the time and date noted on the bag and weighed. Consistency and weight of each stool were assessed and recorded by a paramedical worker who was unaware of either the intervention received by the patient or the intent of the study. This was done as the intervention could not be blinded to the patient or the nursing team because of the distinctive appearance of the two solutions. Assessment of stool consistency was based on the Bristol stool form scale restricted to types V, VI and VII, i.e formed, mushy and liquid stool [16]. Urine output was recorded and monitored. Patients were evaluated at the end of four hours by the study doctor and subsequently every four hours if diarrhea continued or if urine output was not satisfactorily established. Intravenous fluids (Ringer's lactate) were administered by the study doctor if systolic blood pressure lower than 110 mm Hg was recorded following commencement of oral hydration or if the patient had not passed urine within 8 hours after commencement of oral hydration and had a persistently dry tongue. The study nurses and doctors encouraged patients to take ORS according to the schedule prescribed. Serum creatinine and electrolytes were measured at presentation and repeated after 24 hours if normal. In all others, repeat assessment was performed after 4 hours and again at 24 hours. Patients remained in hospital for 48 hours or until the stool consistency was reported as ‘formed’.

**Table 1:** Composition of the two oral rehydration solutions used.

Constituent	HAMS-ORS	HO-ORS
Glucose mM/L	0	75
Na mE/L	75	75
K mE/L	20	20
Cl mE/L	65	65
Citrate mE/L	30	30
HAMS	50 g/L	0
Osmolarity mOsm/kg	170*	245

HO-ORS refers to the currently WHO-recommended hypo-osmolar oral rehydration solution, while HAMS-ORS refers to the same ORS wherein glucose is replaced by amylase-resistant high amylose maize starch (HAMS).

* Excluding products of HAMS fermentation.

### Objectives

The hypothesis was that substitution of amylase-resistant starch for glucose in hypo-osmolar ORS would significantly reduce fecal weight (volume) and diarrhea duration in adults with cholera or cholera-like illness, with at least therapeutic equivalence in hydration. The specific objectives were to conduct a randomized controlled clinical trial in adults with cholera or cholera-like illness using either hypo-osmolar glucose ORS or hypo-osmolar HAMS ORS (i.e. with glucose replaced by high amylose maize starch) and to determine the effect on fecal weight, diarrhea duration, unscheduled intravenous fluid administration, and total ORS intake.

### Outcomes

Primary outcomes were: (1) Duration of diarrhea defined as time from commencing ORS to first formed stool and (2) Total diarrheal fecal weight. Secondary outcome measures included: (1) Fecal weight in the time periods 0–12 hours, 13–24 hours, and 25–48 hours after commencing ORS; (2) Total ORS intake; (3) Need for unscheduled intravenous fluids; and (4) Serum sodium of 134 mE/L or less. The occurrence of elevated serum creatinine (>1.5 mg/dL) at 48 hours and of hypokalemia (serum K <3.5 mmol/L) was also recorded.

### Sample size

The study was designed as a randomized controlled clinical trial. Sample size was calculated using PS Calc [17] based on data in the control group from a previous study in patients with cholera [14]. The aim was to ensure that addition of amylase-resistant starch would reduce duration and weight of diarrhea to an extent that would be considered as clinically significant by the user. Using mean duration of diarrhea of 91 hours (SD 29 hours), and adjusting for multiplicity of analyses, and allowing for 10% dropout, enrolment of 25 patients per study arm would provide 80% power to detect reduction in duration of diarrhea by 28 hours (30% reduction) at the two sided 0.025 significance level. Using the 0–48 hour fecal weight of 12040 g (SD 2751 g), enrolling 25 patients per study arm would provide 90% power to detect reduction in fecal weight by 3010 g (25% reduction) at the two sided 0.025 significance level. Thus, we decided to enroll 50 patients for the study.

### Randomization—Sequence generation

A table of random numbers was computer-generated by randomization such that there was equal distribution among the two groups in fifty participants. Sachets of the appropriate ORS were packaged, as per the random table, in sealed opaque covers bearing serial study numbers.

### Randomization—Allocation concealment

When a participant entered the study, the next available serial number was allocated and the cover bearing this serial number, containing the packaged ORS, was handed by the study physician to the nursing team. The sequence was concealed until the interventions were assigned.

### Randomization—Implementation

The allocation sequence was generated by SV prior to the start of the study. Participants were enrolled by the study medical officers (including VM and BKS) who assigned participants to their groups by handing over the sealed cover bearing the next serial study number (containing the appropriate packaged ORS) to the study nurses.

### Blinding

The participants could not be blinded to the intervention. Those assessing the outcomes were blinded to group assignment. The physicians assessing hydration of the patient were unaware of the intervention received by the patient. The fecal weight was assessed by a para-medical worker who was unaware of the exact nature of the study and did not come into contact with the participants.

### Statistical methods

Survival curves were estimated by the Kaplan-Meier method and compared using the log-rank test. Categorical variables were reported as numbers and the Fisher exact test was used to assess significance of differences between groups. Continuous variables that were not distributed normally were reported as median with interquartile range (IQR) and significance of differences between groups tested using the Mann-Whitney U test. Continuous variables that were normally distributed were reported as mean with standard deviation (SD) and significance of differences between groups was tested using the two-tailed unpaired Student t-test. A Bonferroni correction was used to adjust for the multiple end points as well as multiple time points for fecal weight (i.e., 0–12, 13–24, 25–48 hours). Both unadjusted and adjusted P (P_adj_) values are presented. Two tailed P values less than 0.05 were considered statistically significant. Statistical analysis was performed using InStat for Windows version 3.06 and Prism4 for Windows version 4.03 (GraphPad Software, San Diego California USA).

## Results

### Participant flow

**Figure 1:**
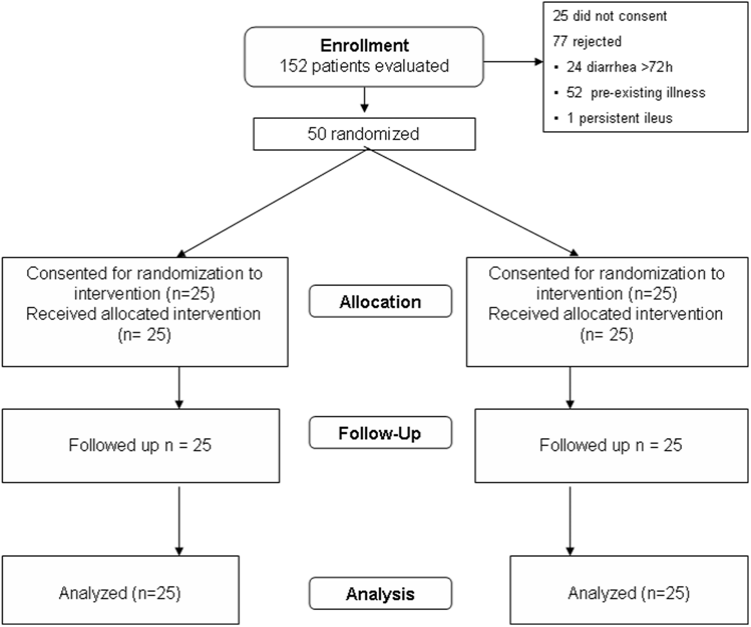
CONSORT flow diagram

50 adult males with acute watery diarrhea and moderate to severe dehydration were included in this study. Figure 1 presents the flow chart for participants enrolled in the study. Patients who were excluded from the study included those with diarrhea duration longer than three days at presentation, and one patient with persistent vomiting and abdominal distension who could not receive oral fluids. Those with pre-existing medical conditions including hypertension and/or diabetes mellitus, renal failure, heart disease, gastrointestinal and liver disease, and internal malignancy, were mostly patients who were visiting this tertiary referral care hospital in south India for medical attention and had developed a diarrheal illness during travel or during their stay in this town.

### Recruitment

Participants were recruited from among consenting patients seen in the Emergency Services between May 2003 and June 2005. During the same period, a total of 913 patients with acute diarrhea were admitted to the hospital.

### Baseline data

The baseline demographic and clinical characteristics of the participants in each group are shown in Table 2. None of the patients included in this study required dialysis for renal failure. There was no mortality in this study.

**Table 2:** Demographic characteristics of patients enrolled in the study.

	HAMS-ORS (n = 25)	HO-ORS (n = 25)
Age (in years) Mean±SD	42.6±12.0	37.8±13.7
Range	24–65	18–63
Diarrhea duration prior to admission (hours)		
Mean±SD	24.4±12.6	27.5±17.7
Range	9–48	2–72
Weight (kG)	62.2±4.7	63.1±5.0
Systolic blood pressure at admission (mm Hg)	75±39.7	70±44.3
Diastolic blood pressure at admission (mm Hg)	37±38.3	39±36.0
Serum creatinine at admission (mg/dL)	1.8±1.1	2.0±0.90
Serum Na at admission (mEq/L)	138.7±6.1	139.2±4.8
Serum K at admission (mEq/L)	4.2±0.53	4.4±0.99
Pathogens isolated		
*V. cholerae* O1	8	11
*V. cholerae* non-O1, non-O139	2	1

Values shown are mean±SD except for pathogens isolated which are actual numbers of patients from whom they were isolated.

### Numbers analyzed

All 50 participants completed the study and their data was analyzed.

### Outcomes and estimation

The time (median, IQR) to first formed stool (duration of diarrhea) was significantly reduced in patients receiving HAMS-ORS (19.0 hours, IQR 10-28) compared to those receiving HO-ORS (42.0 hours, 24–50) (P<0.001, P_adj_<0.001). One patient in the HO-ORS group had prolonged diarrhea lasting longer than four days. The difference between the two groups remained highly significant even when this outlier value was excluded.

**Figure 2:**
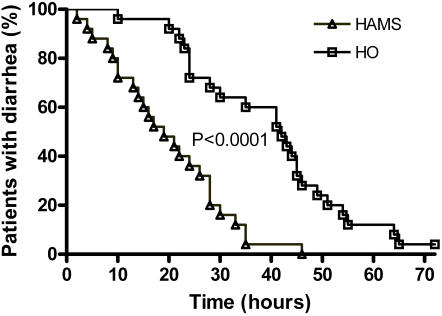
Recovery from diarrhea shown as residual proportion of patients with diarrhea at each time point after commencement of ORS. The difference between high amylose maize-ORS (HAMS) and hypo-osmolar-ORS (HO) was statistically highly significant (P<0.0001, log-rank test).

Survival analysis showed significantly more rapid recovery from diarrhea in the HAMS-ORS group compared to the HO-ORS group (P<0.0001, log rank test) (Figure 2).

**Figure 3:**
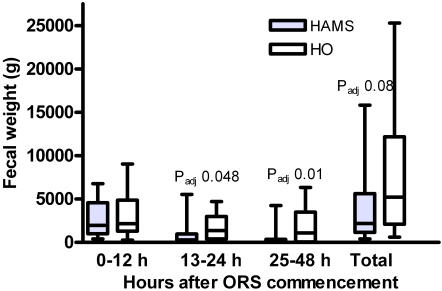
Fecal weight during the time periods 0–12 hours, 13–24 hours, and 25–48 hours after commencement of ORS and total diarrheal fecal weight. The box and whiskers plots show median and IQR (box) and lowest and highest value (whiskers). Two-tailed adjusted P values (Mann-Whitney) show the significance of differences between HAMS and HO ORS for that particular time period.

Total fecal weight (in grams, median, IQR) from entry into the study until the first formed stool was substantially less but not statistically significantly different in the HAMS-ORS group (2190, 1160–5635 g) compared to the HO-ORS group (5210, 2095–12190 g) (P = 0.04, P_adj_ = 0.08) (Figure 3). When analyzed according to the time period when the reduction in stool weight occurred, fecal weight during the first 12 hours of the study was found to be similar in the HAMS-ORS group (1970, 1005–4565 g) compared to the HO-ORS group (2160, 1285–4870 g) (P = 0.51) (Figure 3), whereas in the second 12 hours after commencing ORS fecal weight was significantly lower in the HAMS-ORS group (280, 0–965 g) compared to the HO-ORS group (1360, 405–2985 g) (P = 0.008, P_adj_ = 0.048). Fecal weight in the 24–48 hour study period was also significantly lower in the HAMS-ORS group (0, 0–360 g) compared to that of the HO-ORS group (1080, 55–3485 g) (P = 0.002, P_adj_ = 0.01).

**Figure 4:**
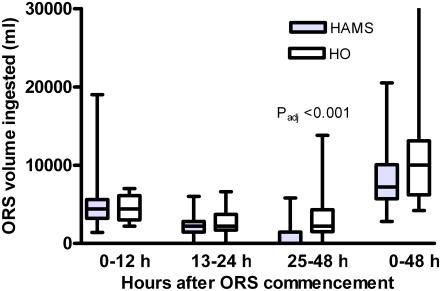
ORS intake in mL during the time periods 0–12, 13–24, and 25–48 hours and total ORS intake (last two box plots). The box and whiskers plots show median and IQR (box) and lowest and highest value (whiskers). Two-tailed adjusted P values (Mann-Whitney) show the significance of differences between HAMS and HO ORS for that particular time period.

ORS intake (median, IQR) in the first and second 12 hours after admission was similar in the HAMS-ORS group (4400, 3200–5600 ml and 2200, 1450–2800 ml) and the group receiving HO-ORS (4400, 3000–6100 ml and 2200, 1700–3700 ml) (P = 0.79 and 0.46 respectively). However, in the second 24 hour period after entry, ORS intake was significantly lower in the HAMS-ORS group (0, 0–1450 ml) compared to the HO-ORS group (2200, 1500–4300 ml) (P_adj_<0.001) (Figure 4). For the entire 48 hour period there was no statistically significant difference of ORS intake in the group receiving HAMS-ORS (7200, 5700–10050 ml) compared to HO-ORS (10000, 6200–13100 ml) (P_adj_ = 0.12).

Administration of intravenous fluids after commencement of ORS was considered necessary in 9 patients randomized to HAMS-ORS compared to 12 patients randomized to HO-ORS (P = 0.56) (Table 3). All these patients required IV fluids within the first 24 hours of admission. There was no significant difference in the amount of fluid required (Table 3).

**Table 3:** Biochemical parameters after therapy and other measures in the two study arms.

	HAMS-ORS	HO-ORS	P
Serum [Na] mEq/L	138.3±4.5	138.5±4.3	0.75
Serum [K] (mEq/L)	4.25±0.58	4.42±0.49	0.30
Serum creatinine (mmol/L)	1.33±0.63	1.31±0.52	0.69
Serum sodium ≤134 mEq/L on admission	4	3	1.00
Serum sodium ≤134 mEq/L at 24 hours	3	2	1.00
Unscheduled IV before 24 h	9 patients	12 patients	0.56
Amount of unscheduled IV fluid given (mL)	1440±490	1210±420	0.47
Amount of water ingested (mL)			
0–12 h	194±382	185±357	0.93
13–24 h	312±437	262±388	0.67
25–48 h	640±795	562±623	0.70
Urine output (mL)			
0–12 h	1477±900	1863±1643	0.31
13–24 h	1729±1156	1285±807	0.13
25–48 h	1798±1469	1940±1149	0.76

Values shown are absolute numbers of patients or mean±SD.

### Ancillary analyses

**Figure 5:**
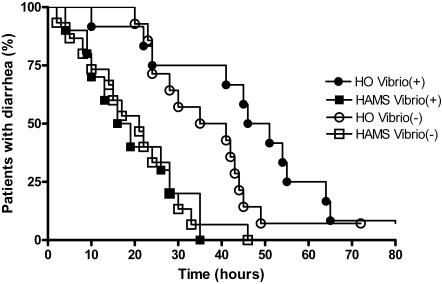
Recovery from diarrhea in patients with positive cultures for *Vibrio cholerae* compared with those with negative cultures. Significant differences were noted between HAMS and HO ORS-treated patients in patients whose stool culture revealed *Vibrio cholerae* (log rank test, P<0.001). Significant differences were noted between HAMS and HO ORS-treated patients also in the group of patients whose stool culture did not grow *Vibrio cholerae* (log rank test, P = 0.006). The *Vibrio*-positive subgroup was not statistically significantly different from the group that was negative by culture, whether it was HAMS or HO-ORS, and the *Vibrio*-positive subgroup showed significantly quicker recovery when treated with HAMS.

Subgroup analysis was performed to compare stool weight and diarrhea duration in patients with positive stool culture for *Vibrio cholerae* compared to those whose stool culture was negative. There was no difference in recovery from diarrhea between *Vibrio* positive and *Vibrio* negative patients. Treatment with ORS type, not the presence of cultivable *Vibrio*, was the determinant of recovery time (Figure 5). In the *Vibrio* positive subgroup, both diarrhea duration and total stool weight were significantly lower in the HAMS-ORS group compared to HO-ORS group (P_adj_ = 0.008 and P_adj_ = 0.025 respectively).

### Adverse events

Hyponatremia (serum sodium less than 134 mEq/L) was noted in 4 patients in the HAMS-ORS group and 3 patients in the HO-ORS group prior to ORS treatment, and in 3 and 2 patients in the respective groups 24 hours after commencement of ORS. None of these patients had any clinical deficit. Four of the 5 patients who had serum sodium of 134 mE/L or less at 24 hours were the same as the ones who had low levels at entry. One patient who received HO-ORS had serum sodium of 136 mE/L at entry and sodium of 128 mE/L at 24 h. There was no significant difference in serum potassium or serum creatinine between the two groups.

## Discussion

The present study demonstrates that diarrhea duration and fecal weights 13–48 hours after start of oral therapy were reduced in cholera and cholera-like diarrhea by treatment with a hypo-osmolar HAMS-containing ORS compared to hypo-osmolar glucose ORS. The findings are particularly important because reduction in diarrhea of the magnitude noted in these studies has not been achieved by hypo-osmolar glucose ORS which at best reduced diarrhea by 20% in children with diarrhea [5] and not at all in adults with cholera [18].

Previous studies suggest that when diarrhea duration is reduced by an intervention, total fecal weight is also reduced. In this study, although there was a substantial decrease in stool output for the entire 48 hours (5210 vs 2190 g), the difference between the two groups did not reach statistical significance primarily because the initial 0–12 hour stool collection did not reveal any decrease (1970 vs 2160 g). Significant differences in fecal weight (which can be perceived as severity of diarrhea) became apparent only after the first 12 hours suggesting that there was a latent period before the action of HAMS-ORS. This supports the hypothesis that the effect of HAMS is secondary to metabolism and absorption in the colon. Two major end points were studied, i.e. diarrheal stool loss and duration of diarrhea. Since there were multiple analyses, a Bonferroni correction was applied, and significant differences were noted between the two groups in diarrhea duration as well as in fecal weight after the first 12 hours. In the prior study of HAMS-ORS using an iso-osmolar ORS solution in patients with cholera [14], a reduction in stool output in the HAMS group was also not observed in the initial 12 hour period consistent with the hypothesis that HAMS is enhancing colonic fluid absorption that will be evident only in later time periods. Attenuation of the mucosal cyclic AMP response by butyrate produced from amylase resistant starch is another possible explanation for the beneficial effect of HAMS and may account for the time lag between HAMS administration and the reduction in diarrhea. The contribution of these and other mechanisms to the HAMS effect remains to be elucidated.

ORS use is based on the concept that absorptive and secretory processes in the intestine and colon are separate and distinct; that stimulation of secretion does not affect absorption; and that enhancement of absorption does not alter secretion. Cyclic AMP induces fluid and electrolyte secretion, but enhances glucose-stimulated sodium absorption [19], and thus glucose ORS enhances sodium and water absorption despite persistence of fluid secretion. In acute diarrhea, the colonic capacity for absorption of water of approximately 5 liters per day [20] is not utilized because of a depletion of luminal short chain fatty acids (SCFA) [21]. High amylose maize starch (HAMS) contains a mixture of digestible and amylase-resistant starches. The digestible component provides glucose in the small intestine for facilitating sodium absorption. The amylase-resistant component results in an increase of colonic luminal concentrations of SCFA [22]. SCFA production in the colon is difficult to measure because of the extremely rapid absorption and metabolism leading to their disappearance from colonic lumen and feces, and is usually inferred indirectly from the amount of substrate administered and the amount recovered from feces. We have previously demonstrated that fecal recovery of HAMS administered into the stomach of patients with cholera was only 16% [14], indicating that over 80% of the administered amylase resistant starch had been utilized, presumably by fermentation to SCFA in the colon. It must be noted that standard therapy for cholera also included the early introduction of normal food which included rice and other cereals which can also provide substrate for colonic fermentation to short chain fatty acids. This intervention probably contributed to the beneficial effect in the HAMS group by leading to an increased colonic carbohydrate load in the latter patients. In the present study a single oral dose of doxycycline was administered as part of standard clinical practice for the treatment of cholera. Administration of an antibiotic, to which the cholera Vibrio is sensitive, has been shown to significantly shorten diarrhea [23]. *Vibrio cholerae* isolates from southern India are presently sensitive to tetracycline although isolates from several parts of the world exhibit tetracycline-resistance. It has been shown that the anaerobic bacterial flora of the colon, which is responsible for starch fermentation to SCFA, is not affected by oral doxycycline [24].

Participants in this study had severe acute watery diarrhea with significant dehydration at presentation, necessitating initial intravenous rehydration. Although fecal culture was positive for *Vibrio cholerae* only in 22 of these 50 patients, in southern India, severely dehydrating acute infective diarrhea in adults is almost always due to cholera. Such diarrhea may also be caused by enterotoxigenic *E. coli* that elaborate heat labile enterotoxin and produce a syndrome similar to cholera [25]. Standard bacterial cultures did not reveal the presence of any other bacterial enteropathogens in these patients, nor were parasites noted in any of the patients; however we did not test for enterotoxigenic *E. coli*. When sub-group analysis was done on those patients with positive stool cultures for *Vibrio cholerae*, the difference between the two treatment groups remained statistically highly significant. In an earlier study, our group demonstrated that addition of HAMS to conventional (iso-osmolar) glucose ORS reduced diarrhea duration and fecal weight in patients with cholera [14]. Children with non-cholera diarrhea possibly represent a different group by virtue of the different spectrum of pathogens that cause diarrhea. The ability of colonically-absorbed carbohydrate to shorten diarrhea in children has been examined with variable results. HAMS and partially hydrolysed guar gum both shortened diarrhea compared to conventional iso-osmolar glucose ORS [26], [27]. However, a mixture of colonically-absorbed carbohydrates did not shorten diarrhea when compared to reduced osmolarity glucose ORS [28]. The latter finding can be ascribed either to the small amount of colonically-absorbed carbohydrate that was added or to the comparison with a reduced osmolarity ORS which may already have maximally increased absorption. The present study is different in that it examined the possibility that HAMS could reduce diarrhea in adult patients with cholera treated with a low sodium hypo-osmolar ORS.

The large volume of ORS given in both trial arms resulted from following the recommendation to administer 200 ml of ORS every hour in addition to 200 ml after each loose stool, until such time as the stool became formed. This large volume of ORS led to increased urine volume in both groups. Although not to be recommended as physiological, the apparently excessive ORS administration and urine volume served to protect against acute renal failure which remains a significant complication in patients with acute severely dehydrating watery diarrhea. Despite the excessive ORS administration, ORS intake in the period 25–48 hours after commencement of treatment was significantly less in the HAMS-ORS group (median 0 ml) compared to the HO-ORS group (median 2200 ml). It is possible that total ORS intake would have been much less in both groups had intake been exactly matched to stool output. Thus, as earlier mentioned in connection with fecal volumes, the methodology used could possibly have confounded the outcomes by affecting the parameters measured.

Concerns have been raised about the possibility of symptomatic hyponatremia in patients with cholera treated with HO-ORS [29]. In the present study, we did not observe symptomatic hyponatremia in any of the patients. However, the numbers of patients included in this study were small. In order to be powered to detect significant hyponatremia in any treatment arm, the study would have had to include a much larger number of participants, but this was not the primary study aim. The rates of unscheduled intravenous infusion in this study were higher than in other studies of oral rehydration. The overall failure rate of oral rehydration in community practice is considered to be in the range of 1%. However, this hospital-based study recruited patients with severe diarrhea, and supplemental intravenous fluids were given in patients who failed to pass urine within the first eight hours after completion of IV hydration in order to ensure that they would not develop renal failure. The need for such IV fluid administration was equal in the two study arms. The administration of fixed quantities of oral rehydration solution in this study, instead of replacing stool volume with equivalent amounts of ORS, may also have led to a higher incidence of unscheduled intravenous fluid administration.

In designing a new ORS that would reduce stool weight and diarrhea duration, we felt that it would be important for the difference to be not only statistically significant but also both quantitatively appreciable and clinically substantial. In the present study, substitution of glucose in hypo-osmolar ORS by high amylose maize starch reduced the duration of diarrhea by 55% and very significantly reduced fecal weight after the first 12 hours of illness. Hypo-osmolar ORS has become the recommended standard of therapy for the oral therapy of cholera. However in patients with cholera, hypo-osmolar ORS does not reduce fecal weight or shorten diarrhea [6]. Cholera and similar severe watery diarrhea often occurs in resource-poor countries or in refugee camps. In such situations, especially when it occurs in epidemics, the use of ORS containing HAMS may be of particular benefit in shortening diarrhea, hospitalization, and reducing costs.

## Supporting Information

Protocol S1 Trial Protocol
(0.04 MB DOC)

Checklist S1 CONSORT Checklist
(0.05 MB DOC)
