# Supplementary material for: A Randomized Controlled Trial of Glucose versus Amylase Resistant Starch Hypo-Osmolar Oral Rehydration Solution for Adult Acute Dehydrating Diarrhea
Source: PLoS One. 2008 Feb 13;3(2):e1587. doi: 10.1371/journal.pone.0001587 (PMC2217593; doi:10.1371/journal.pone.0001587)
Supplement: Protocol S1 — Trial Protocol (0.04 MB DOC) [file pone.0001587.s001.doc]

**AIM**:

To determine whether high-amylose maize starch (HAMS) in hypo-osmolar (reduced osmolarity) ORS will reduce stool output and lessen duration of diarrhoea in patients with cholera or cholera-like illness, when compared to glucose-containing hypo-osmolar (reduced osmolarity) ORS.

**SUBJECTS**:

Inclusion Criteria:

1. Males aged between 12 years to 65 years.
2. Profuse watery diarrhoea of 3 days duration or less.
3. Moderate to severe dehydration at presentation.

Exclusion Criteria:

1. Bloody diarrhoea.
2. Concurrent major medical illness including cardiac, renal or liver disease or internal malignancy.

**STUDY PROTOCOL**:

1. Patients will be recruited from the Emergency Services (Casualty) of the Christian Medical College & Hospital, Vellore.
2. The Casualty Medical Officer (CMO) will page the study medical research officer when male patients with acute watery diarrhoea and moderate to severe dehydration present to Emergency Services.
3. The CMO will resuscitate the patient, placing a wide bore peripheral or central venous line as per clinical judgement, and running in intravenous Hartmann’s Ringer lactate at a total dose of 100 ml/kg as per WHO treatment guidelines.
4. Blood samples will be collected for blood counts, urea, creatinine and electrolytes.
5. The nurse will send stool samples for culture (*Vibrio cholerae* and enteric bacterial pathogens) as soon as feasible.
6. A single oral dose of Doxycyline 300 mg will be given.
7. The study medical officer will evaluate the patient for eligibility and obtain informed written consent from patient or relative if eligible.
8. The study medical officer will re-evaluate the patient four hours after commencing intravenous rehydration in order to confirm that the patient can enter the trial.
9. Eligible patients who have been consented will be transferred to the gastroenterology metabolic ward for further treatment.
10. On reaching the ward, the study medical officer and nurses will evaluate the patient and again explain the details of the study including the need to take oral rehydration solution, the manner in which it is to be taken, and details regarding food intake, stool collection and urine collection.
11. Each patient that enters the ward and is ready to receive ORS will be given a packet labeled with the next serial study number and containing 50 sachets of ORS (for reconstitution to 200 ml each).
12. Serial study numbers will be randomly allocated to one of the two ORSs being studied using a table of random numbers, and the code retained with the study co-ordinator in a sealed envelope. Sachets of the two ORSs will be packaged accordingly. Backup packets with additional ORS sachets will be available for each study number.
13. Patients will be provided a plastic measuring cylinder for making up the ORS. They will be advised to use bottled water for making up the ORS. They will be instructed to take 200 ml ORS per hour and 200 ml after each loose stool. The ORS will be mixed and administered by the study nurse or by the patient’s attendant.
14. Patients will be provided bottles for urine collection, and buckets lined with plastic bags for stool collection. A chart for recording time of each stool, as well as the consistency of each stool will be given to each patient and the attendant of the patient will be instructed to complete this.
15. A normal south Indian diet will be provided to the patient and he will be encouraged to take this while ORS administration is continuing.
16. One study nurse will be constantly available, by rotation, to ensure that the patient receives ORS and collects stool and urine quantitatively.
17. The study medical officer will review the patient every four hours or more frequently in the first day, in order to ensure that hydration is maintained and renal function is not compromised.
18. As it is difficult to mask the starch containing ORS, the study medical officer should avoid asking about the kind of ORS which the patient is receiving.
19. Stool collected in individual labeled plastic bags will be taken to the faecal laboratory by the ward attender and left in a designated place. These bags will be weighed periodically by the paramedical worker assigned to the study team, who will record the weight and consistency and then send the bags for incineration. This paramedical worker will not come into contact with the patient or study nurses and will have no knowledge of the intervention received or the actual intent of the study.
20. Urine output will be recorded with time of voiding.
21. Serum creatinine and electrolytes will be measured after 6-12 hours in patients whose urine output or renal function is not satisfactory, and after 24 hours in all patients.
22. In any patient whose dehydration is not adequately corrected (as evidenced by clinical signs of dehydration including a persistently low urine output) or who continues to have profuse diarrhoea exceeding 10 ml/kg/hour, intravenous fluids may be administered according to the clinical judgement of the medical officer supervising the study. All fluid intake is to be recorded including such unscheduled intravenous fluid administration.
23. Patients will be discharged after 48 hours or when their diarrhoea has ceased, whichever occurs later.
24. In an individual patient, the study will end at the point when the first formed stool is observed. This time must be noted for each patient.
25. The major or primary **end-points** of the study are:
26. Stool weight in the first and second 12 hour periods
27. Stool weight in the second 24 hours
28. Time from first ORS administration to first formed stool

26. Other or secondary end-points include:

1. Amount of ORS consumed during 0-24 and 24-48 hours
2. Incidence of unscheduled IV treatment in the two study arms
3. Incidence of complications – hypokalaemia (serum potassium <3.5 mE/L), hyponatraemia (serum sodium <130 mE/L).
